# Effect of Aging on Hedonic Appreciation of Pleasant and Unpleasant Odors

**DOI:** 10.1371/journal.pone.0061376

**Published:** 2013-04-24

**Authors:** Pauline Joussain, Marc Thevenet, Catherine Rouby, Moustafa Bensafi

**Affiliations:** CNRS UMR5292, INSERM U1028, Lyon Neuroscience Research Center, University Lyon, Lyon, France; Duke University, United States of America

## Abstract

Does hedonic appreciation evolve differently for pleasant odors and unpleasant odors during normal aging? To answer this question we combined psychophysics and electro-encephalographic recordings in young and old adults. A first study showed that pleasant odorants (but not unpleasant ones) were rated as less pleasant by old adults. A second study validated this decrease in hedonic appreciation for agreeable odors and further showed that smelling these odorants decreased beta event-related synchronization in aged participants. In conclusion, the study offers new insights into the evolution of odor hedonic perception during normal aging, highlighting for the first time a change in processing pleasant odors.

## Introduction

One important aspect of olfaction is its salient affective dimension that can entail withdrawal or approach behaviors [1], [2]. First, a particular odor can provide an early warning signal against toxic substances (spoiled or toxic food, industrial pollutants), enabling such dangerous substances to be avoided. Second, olfaction plays a major role in hedonic pleasure. Positive affects evoked by food or flowers demonstrate how olfaction can make our life more pleasant. Evidence of the existence of two different systems dedicated to treating aversive and appetitive chemosensory stimuli has been provided by psychophysical and neuroimaging studies showing that unpleasant odors are processed faster than pleasant ones [3], [4], [5], inducing specific patterns of autonomic [6], [7] and olfactomotor responses [8], [9], [10] and specific neural activation [11], [12], [13], [14], [15], [16].

How these two opposite facets of hedonic responses to odors evolve with age remains unclear today. Indeed, studies of the emotional perception of odors during aging are rare and not consensual: in one study, the smells of lavender and spearmint [17] were judged more pleasant by the older subjects, while another study [18] found such an age effect for certain smells (turpentine, garlic and fish became less unpleasant with age and cloves and rose more pleasant) but not for others (orange, leather, cinnamon, spearmint, banana, lemon, anise, coffee, apple, pineapple, licorice). In contrast, by taking into account the hedonic valence of the odor, another study suggested that older subjects exhibit decreased identification abilities of pleasant but not unpleasant odors [19]. The main aim of the present study was therefore to investigate how hedonic appreciations of both pleasant and unpleasant odors evolve with age by using psychophysical and neurophysiological methods.

First, the effect aging on the hedonic appreciation of pleasant and unpleasant odors was tested (Experiment 1). To this end, the performances of a group of young and a group of older subjects were compared in a task consisting in smelling pleasant and unpleasant odorant molecules and providing hedonic appreciation for each. Here, intensity ratings were also collected.

Second, we set out to examine the effect of aging on both perceptual and neural processing of pleasant and unpleasant odors (Experiment 2). To this end, psychophysical and neurophysiological responses to pleasant and unpleasant odors were assessed in 2 groups of young and of older adults. Neurophysiological studies based on olfactory event-related spectral perturbation (OERSP) showed that odors induced significant beta-type and low-gamma-type oscillations in the amygdala of epileptic patients [20] and theta-type and alpha-type oscillations in healthy subjects [21]. Moreover, the latter study revealed a positive correlation (r = 0.70) between olfactory capabilities (detection, discrimination and identification) and event-related synchronization in the theta band. Given this strong relationship between oscillatory responses and olfactory perception, OERSPs were recorded in the present study. Because the effect of odor hedonic valence on OERSP was never estimated to the best of our knowledge, we examined the influence of aging on event-related synchronization in the above 4 frequency bands (from theta to low-gamma) during exposure to pleasant and unpleasant odors.

## Results

**Figure 1:**
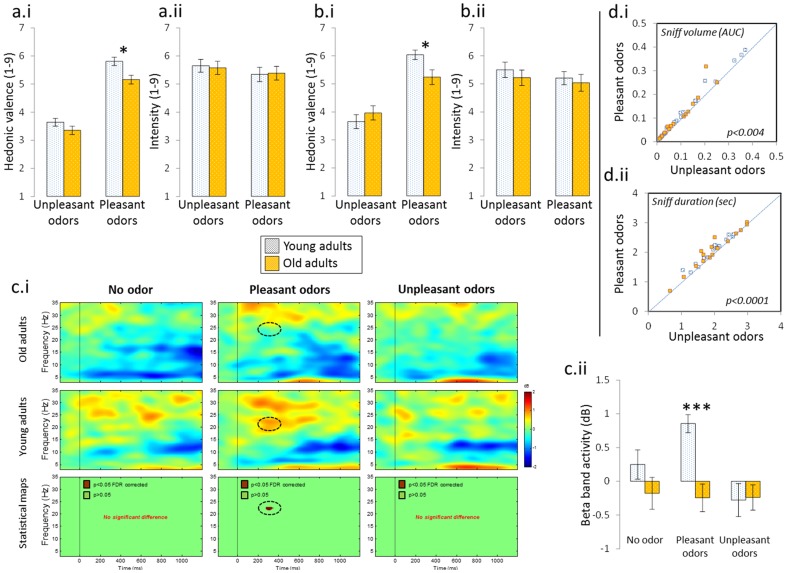
Effects of aging on hedonic appreciation and EEG responses to pleasant and unpleasant odors. (a) Experiment 1. Hedonic appreciation (a.i.) but not intensity ratings (a.ii.) of pleasant odors decreased in old adults. (b) Experiment 2 - psychophysics. Hedonic appreciation (b.i.) but not intensity ratings (b.ii.) of pleasant odors decreased in old adults. (c) Experiment 2 – OERSP. Time-frequency maps for the “no odor”, “pleasant odors” and “unpleasant odors” conditions in old adults and young adults, and corresponding statistical maps (c.i.). A significant difference between Old adults and Young adults was observed for pleasant odors, at the corrected statistical threshold of p<0.05 (FDR). Beta event-related synchronization decreased in old adults only for pleasant odors (c.ii.). (d) Experiment 2 – sniffing. Sniff volume (d.i.) and sniff duration (d.ii.) are significantly greater for pleasant odors than for unpleasant odors, both in young (white square) and old (orange square) adults. Error bars correspond to SEM. * = p<0.05, *** = p<0.0001.

In Experiment 1, for pleasantness ratings, an effect of “Age” (F[1.36] = 6.693, p = 0.01) associated to an effect of “Hedonic valence” (F[1,36] = 370.990, p<0.0001), and a nearly significant “Age-by-Hedonic valence” interaction (F[1,36] = 3.039, p = 0.08) were observed, suggesting that whereas no significant effect of “Age” occurred for unpleasant odors (p>0.05), older adults rated pleasant odors as less pleasant than did young adults (p = 0.004) (Fig. 1a.i). For intensity ratings, although a trend toward an effect of intensity on “Hedonic valence” was observed (F[1,36] = 4.101, p = 0.0503), no “Age” effect (F[1,36] = 0.002, p>0.05) or “Age-by-Hedonic valence” interaction (F[1,36] = 0.234, p>0.05) were significant (Fig. 1a.ii). To sum up, the hedonic appreciation of pleasant odors decreased in older participants. This effect could not be explained by changes in perceived intensity.

In Experiment 2, the ANOVA on pleasantness ratings revealed no effect of “Age” (F[1,31] = .784, p>0.05) but a significant effect of “Hedonic valence”(F[1,31] = 97.087, p<.0001) accompanied by a significant “Age-by-Hedonic valence” interaction (F[1,31] = 8.880, p = 0.005), reflecting the fact that whereas no significant effect of “Age” occurred for unpleasant odors (p>0.05), older adults rated pleasant odorants as less pleasant than did young adults (p = 0.014) (Fig. 1b.i).

For intensity ratings, an effect of “Hedonic valence” was seen (F[1,31] = 6.487, p = 0.016) but was not accompanied by any effect of “Age” (F[1,31] = 0.367, p>0.05) or “Age-by-Hedonic valence” interaction (F[1,31] = 0.349, p>0.05)(Fig. 1b.ii).

Analysis of OERSP data revealed that odorants induced changes in oscillatory response in various frequency bands (from theta to low-gamma) in all electrode sites. However, statistical analysis revealed a significant effect of “Age” in P4 for pleasant odors (p<0.05 FDR corrected) in the beta band (20–25 Hz) between 250 and 400 ms following odorant presentation, reflecting a decrease in event-related synchronization in the beta band for pleasant odors in older compared to young adults (Fig. 1c.i). No such significant effect of “Age” was observed for the no odor control condition and for the unpleasant odors.

Based on this observation, and as in the study of Huart et al [21], we defined a time-frequency ROI in this EEG band (275–375 ms centered at 22 Hz). Results revealed an effect of “Age” (F[1.31] = 6.673, p = 0.01) associated to an effect of “Conditions” (F[2,62] = 4.330, p = 0.02) (here “conditions” included 3 modalities: “no odor”, “pleasant”, “unpleasant”), and a significant “Age-by-Conditions” interaction (F[2,62] = 4.457, p = 0.02). Mean comparisons showed that whereas no significant effect of “Age” occurred for the no odor condition (p>0.05) and the unpleasant odors condition (p>0.05), a decrease in event-related synchronization in the beta band in older compared to young adults was observed for pleasant odors (p = 0.00008) (Fig. 1c.ii).

To determine whether the above age effects on OERSP were accompanied by any change in sniffing, sniff duration and volume were analyzed in separate ANOVAs according to “Age” and “Hedonic valence”. For sniff duration, a significant effect of “Hedonic valence” (F[1,31] = 19.401, p<0.0001) was observed, but was not accompanied by any effect of “Age” (F[1,31] = 0.037, p>0.05) or “Age-by-Hedonic valence” interaction (F[1,31] = 0.032, p>0.05) (Fig. 1d.i). For sniff volume, here again, a significant effect of “Hedonic valence” was seen (F[1,31] = 9.782, p = 0.004), but was not accompanied by an effect of “Age” (F[1,31] = 2.021, p>0.05) or by a “Age-by-Hedonic valence” interaction (F[1,31] = 0.158, p>0.05) (Fig. 1d.ii).

To sum up, as in previous studies, sniff duration and sniff volume correlated with the pleasantness of the odor in both young adults and older adults [8], [9], [10], but they did not explain the observed effect of aging on EEG activity to pleasant odors.

## Discussion

The present study set out to examine whether aging influences hedonic appreciation of pleasant odors and unpleasant odors differently. The first result of interest was that the hedonic appreciation of pleasant odors decreased in older compared to young adults. This finding was replicated in two successive experiments and could not be explained by perceived intensity effects. Past studies have shown diverging results regarding this topic [17], [18], [19], [22], and our study using a large number of odorants in the same intensity range offers new insights into this non-consensual issue highlighting for the first time a modulation in odor hedonic valence specifically for pleasant odors in old adults.

The second result of interest was that influence of aging on processing of pleasant odors was reflected not only in explicit perceptual ratings but also in the EEG responses: pleasant odors decreased event-related synchronization in the beta band in older individuals. In humans, the significance of EEG rhythms in the olfactory modality is not well known. Recently, Huart et al., have shown a positive correlation between olfactory abilities (detection, discrimination and identification) and event-related synchronization in the theta band. The absence of theta modulation in our case may be due to methodological differences between studies including the populations or the number and type of odors used (pleasant and unpleasant odors are used in our study). Regarding the beta-band, very few knowledge is available in the literature. Even in non-chemosensory modalities, the functional role of beta-band oscillations is not well understood.

In a recent review paper, Engel and Fries [23] proposed that cognitive tasks involving a top-down component should be associated with enhanced beta band activity. Interestingly, olfaction studies suggest that access to lexico-semantic representations is more efficient for pleasant odors: pleasant odors are described using more semantic sources or verbal labels than unpleasant odors [24], [25]. Such top-down processing linking positive smells and semantic memory may explain the larger beta event-related synchronization in response to pleasant odors in young adults. In contrast, in old adults, the access to semantic representations of odors being difficult [26], beta event related synchronization decreased.

We acknowledge that this hypothesis is speculative at this stage and further work is needed to test it, especially by experimental settings including assessments of odor familiarity and edibility, two dimensions that may be altered during normal aging and may explain part of the present finding.

That the hedonic appreciation of pleasant odors is modulated with age is a novel finding. The question arises as to the mechanism of this effect. One possibility may be changes in central brain areas involved in odor processing. These changes in the neural processing of odors may reflect for example differential involvement of primary olfactory structures as a function of both age and hedonic valence since 1) primary olfactory structures such as piriform cortex encode information about hedonic valence [27], [28], and 2) piriform cortex is less activated in older than younger subjects [29]. That the modulation in the beta band is observed at an early stage of processing (around 300 ms) renders possible a modulation at the level of primary olfactory cortex areas.

Another possibility could involve gene expression, which varies across individuals [30] and may show dysregulation during normal aging. Besides olfactory receptors, gene dysregulation could also affect axon targeting of the glomeruli in the olfactory bulb, as the number of glomeruli is reduced in the aged human olfactory bulb [31].

However, a peripheral change as a function of age in the composition of the nasal mucus cannot be excluded. This was not tested in old individuals, but perireceptor changes were postulated in mice for certain odorants sharing ester functional groups, which are targets for metabolic enzymes secreted in the mucus, resulting in fast conversion to the corresponding acids and consequent qualitative change in perception [32].

Interestingly, among all odorants used in the current study, most of the ester molecules were rated as pleasant by young adults (5 esters were pleasant and only one ester was unpleasant). This pattern of results is in line with previous psychophysics experiments suggesting a link between physicochemical properties and odor hedonics [33], [34], [35], [36], especially those showing that esters are usually perceived as more pleasant than sulfur or nitrogen-containing compounds and light carboxylic acids [37]. Moreover, on a neural level, imaging studies demonstrated that odorant molecules differing in functional group (alcohols, esters, etc.) are processed by different networks in the olfactory bulb [38], [39] and in primary olfactory cortex [27], [28]. The question whether these networks show different patterns of evolution during aging is of most interest and could be addressed in future investigation.

While the present study provides evidence that aging influences hedonic appreciation of pleasant odors, some of the experimental choices require discussion.

An olfactometer could have been useful in Experiment 2, in which EEG responses to odors were recorded. However, as in previous neuroimaging [40], [41], [42], [43], [44], EEG [45], [46], [47]and stereo-EEG studies [20], [48], [49], we favored odorant flasks instead of automated stimulation, for 2 main reasons. First, the same stimulus presentation mode was implemented in both experiments, to enable comparisons. The second reason concerned the number of odorants to be tested: an olfactometer would not allow 20 odorants to be tested; most olfactometers used in the field of EEG odor response diffuse only 2 to 6 different stimuli [50], [51], [52]. Having made this choice, accurate time-locking across trials was ensured by recording sniffing during the experiment (to define the onset of each EEG trial) and a significant effect on EEG recordings was observed.

Notwithstanding the above limitations, the present study offers new insights into odor perception during normal aging, highlighting for the first time an influence of aging on hedonic appreciation of pleasant odors, opening up new perspectives on the impact of age on perception of foods or fragrances, with its possible consequences for nutrition and quality of life. In all species including humans, odors are potentially related to acts in the sense that the information they carry is generally used to decide what action to take: move away or approach the odor source. Our study suggests that it is the positive hedonic response to odors that is dampened during normal aging, avoidance behaviors being spared, survival outweighing pleasure.

## Materials and Methods

### Ethics Statement

The experimental procedures of the two experiments were explained in great detail to the subjects, who provided written consent prior to participation. The study was conducted according to the Declaration of Helsinki and was approved by the ethical committee of Lyon Sud-Est. Exclusion criteria were: abnormal olfaction, history of neurological disease or injury, or history of nasal insult (broken nose or surgery).

### Experiment 1

#### Subjects

Thirty-eight participants were tested (19 young adults, mean age = 20.84+/−2.14 yrs; 19 older subjects, mean age = 59.79+/−2.99 yrs).

#### Odorants

The following 25 odorants were used (odorant code, CAS, and volume/volume percentage dilution are given in brackets): Amyl Butyrate (ABU; 540-18-1; 0.51%), Acetophenone (ACE; 98-86-2; 0.56%), Allyl Caproate (ALC; 123-68-2; 0.55%), Amyl PhenylAcetate (APA; 102-19-2; 59.13%), Benzyl Acetate (BEA; 140-11-4; 1.46%), Carvone-l (CAR; 99-49-0; 2.37%), 1-Decanol (DEC; 112-30-1; 33.74%), Dodecanal (DOD; 112-54-9; 27.74%), Diphenyl oxide (DPO; 101-84-8; 13.55%), Ethyl Butyrate (ETB; 105-54-4; 0.01%), Eugenol (EUG; 97-53-0; 13.12%), Geraniol (GER; 106-24-1; 21.25%), Guaiacol (GUA; 90-05-1; 2.08%), Heptanal (HAL; 111-71-7; 0.07%), Heptanol1 (HOL; 111-70-6; 0.91%), Hexanoic Acid (HEX; 142-62-1; 3.63%), 3-Hexanol (XOL; 623-37-0; 0.08%), Ionone-beta (ION; 14901-07-6; 30.60%), Isoamyl Acetate (ISO; 123-92-2; 0.03%), Methyl Anthranilate (MAN; 134-20-3; 12.65%), Phenyl Ethanol (PEA; 60-12-8; 2.66%), Santalol (SAN; 115-71-9; 100%), Thioglycolic Acid (THA; 68-11-1; 0.32%), Trimethyl Amine (TMA; 75-50-3; 0.0001%),IsoValeric Acid (IVA; 503-74-2; 0.19%). All odorants (Sigma-Aldrich®) were diluted in mineral oil so as to achieve an approximate gas-phase partial pressure of 1 Pa. They were presented in 15 ml flasks (opening diameter: 1.7 cm; height: 5.8 cm; filled with 5 ml oil) and were absorbed on a scentless polypropylene fabric (3×7 cm; 3 M, Valley, NE, USA) to optimize evaporation and air/oil partitioning.

#### Experimental procedure

Subjects were seated in a well-ventilated air-conditioned room.

Once instructions had been read and the consent form signed, the experiment started. Each odorant was presented once. For each trial, subjects received task instructions generated by a digitally recorded voice, via speakers. Each trial began with an auditory primer “Please prepare to sniff,” followed by a countdown (“3, 2, 1, sniff”). Odorants were randomly presented by the experimenter 1 cm below the subject's nose for 3 seconds. Subjects were asked to sniff for the full duration of odorant presentation. Inter-trial interval was 45 seconds. In order to habituate the subject to the experimental setting, a training session with a sequence of 1 to 3 empty flasks was carried out. After presentation of each odorant, subjects were instructed to rate hedonic valence and intensity on a scale from 1 (not at all intense/pleasant) to 9 (very intense/pleasant).

#### Data analysis

To examine the influence of aging on hedonic appreciation of pleasant and unpleasant odors, odorants were grouped into two classes as a function of their hedonic valence: 12 odorant molecules were classified as pleasant (SAN, ION, XOL, DEC, ACE, ETB, MAN, ALC, ISO, CAR, HOL, PEA) and 12 odorant molecules were classed as unpleasant (IVA, HEX, BEA, TMA, DOD, APA, THA, GUA, DPO, GER, ABU, HAL). Odorants were put in one of the two groups on the basis of young adult subject's hedonic ratings (one odorant, EUG, which had the most neutral hedonic rating, was not taken into account in the analysis). For each subject, ratings of pleasantness and intensity were then averaged for all odorants of a given category (pleasant and unpleasant) and served as a dependent variable in an analysis of variance (ANOVA) with “age” as a between-subjects factor (2: Young, Older) and “Hedonic valence” as a within-subject factor (2: pleasant, unpleasant). The same statistical analysis was done for intensity ratings. Significant level was set at p<.05 and all statistical analyses were performed using StatView® software.

### Experiment 2

#### Subjects

Thirty-three participants were tested (17 young adults, mean age = 24.2+/−4.1 yrs; 19 older subjects, mean age = 63.5+/−5.0 yrs).

#### Odorants

Because the EEG experiment was longer in duration, we used only 20 odorants in Experiment 2. ABU and IVA were discarded because of their respectively low and high levels of intensity. THA, SAN and TMA were removed randomly. The following 20 odorants used in Experiment 1 were retained: ACE, ALC, APA, CAR, DEC, DOD, DPO, ETB, EUG, GER, GUA, HAL, HOL, HEX, XOL, ION, ISO, MAN, PEA, BEA. It is worth noting that pleasantness scores of the 20 odorants correlated positively between the two experiments (r = 0.44, p = 0.05), with the exception of one odorant (BEA) which was rated more intense and subsequently, more unpleasant in Experiment 1 compared to Experiment 2 (see Table 1). When this odorant was removed from the comparison, the correlation reached a r value of 0.71 (p = 0.0006).

**Table 1:** Olfactory ratings.

		Experiment 1				Experiment 2			
		Pleasantness		Intensity		Pleasantness		Intensity	
		Young	Old	Young	Old	Young	Old	Young	Old
ABU	mean	4.53	4.32	2.89	3.68	-	-	-	-
	sem	0.33	0.39	0.37	0.55	-	-	-	-
ACE	mean	5.63	5.42	6.05	5.84	5.59	5.00	6.38	5.96
	sem	0.30	0.51	0.43	0.42	0.45	0.44	0.30	0.34
ALC	mean	6.05	5.89	4.37	4.68	5.80	4.96	4.06	3.53
	sem	0.19	0.31	0.39	0.51	0.31	0.27	0.30	0.33
APA	mean	3.53	3.05	6.47	6.89	2.87	3.24	6.71	5.68
	sem	0.39	0.40	0.44	0.35	0.44	0.42	0.29	0.41
BEA	mean	2.95	1.79	6.16	6.79	6.60	5.74	4.79	5.03
	sem	0.50	0.24	0.40	0.48	0.20	0.27	0.31	0.39
CAR	mean	6.11	5.79	5.16	5.95	6.93	6.24	5.41	5.03
	sem	0.32	0.46	0.36	0.40	0.32	0.36	0.34	0.41
DEC	mean	5.47	5.84	4.63	4.00	3.57	3.65	5.56	4.95
	sem	0.32	0.35	0.42	0.35	0.31	0.27	0.32	0.36
DOD	mean	3.53	3.79	5.42	4.74	3.46	3.49	5.78	5.56
	sem	0.32	0.28	0.42	0.46	0.28	0.39	0.35	0.38
DPO	mean	4.42	4.47	6.21	5.84	4.06	4.01	4.83	5.45
	sem	0.39	0.45	0.42	0.32	0.31	0.31	0.44	0.31
ETB	mean	5.79	4.00	5.58	5.00	6.19	4.81	4.81	4.63
	sem	0.39	0.43	0.40	0.39	0.49	0.43	0.37	0.41
EUG	mean	5.05	4.53	6.32	6.11	3.75	4.01	6.43	6.29
	sem	0.48	0.45	0.38	0.36	0.38	0.51	0.31	0.35
GER	mean	4.47	4.00	6.42	5.42	5.94	5.84	5.72	5.43
	sem	0.40	0.34	0.40	0.38	0.27	0.43	0.33	0.41
GUA	mean	4.05	3.58	6.42	5.79	3.59	3.31	6.79	6.75
	sem	0.45	0.38	0.42	0.45	0.43	0.45	0.38	0.36
HAL	mean	4.79	4.42	4.32	4.26	3.87	4.49	5.10	3.88
	sem	0.26	0.42	0.48	0.49	0.25	0.33	0.29	0.31
HOL	mean	6.16	5.84	5.74	5.16	4.47	4.06	5.32	4.69
	sem	0.41	0.32	0.43	0.36	0.32	0.42	0.32	0.37
HEX	mean	2.21	2.16	6.53	6.74	2.42	2.64	5.10	5.54
	sem	0.21	0.34	0.49	0.48	0.28	0.20	0.33	0.48
XOL	mean	5.47	4.89	3.58	4.89	4.49	4.88	3.39	2.91
	sem	0.25	0.38	0.38	0.34	0.27	0.24	0.45	0.38
ION	mean	5.37	4.42	5.37	4.74	4.97	4.63	5.14	4.98
	sem	0.36	0.32	0.48	0.45	0.43	0.40	0.32	0.37
ISO	mean	6.11	4.89	5.84	6.63	7.12	5.96	5.66	5.55
	sem	0.41	0.57	0.43	0.41	0.20	0.28	0.27	0.40
MAN	mean	6.05	4.95	6.21	6.47	5.24	5.20	4.58	4.93
	sem	0.33	0.44	0.46	0.34	0.36	0.45	0.36	0.37
PEA	mean	6.26	5.21	5.74	5.37	5.96	5.88	5.52	4.93
	sem	0.30	0.42	0.45	0.41	0.39	0.36	0.22	0.32
SAN	mean	5.21	4.74	5.84	5.89	-	-	-	-
	sem	0.39	0.42	0.41	0.37	-	-	-	-
TMA	mean	3.37	3.16	4.32	4.58	-	-	-	-
	sem	0.30	0.41	0.48	0.56	-	-	-	-
THA	mean	4.00	3.68	5.89	5.37	-	-	-	-
	sem	0.46	0.38	0.23	0.35	-	-	-	-
IVA	mean	1.84	1.79	6.74	6.79	-	-	-	-
	sem	0.29	0.20	0.42	0.52	-	-	-	-

Means and standard errors of the mean (sem) of pleasantness and intensity in young and older adults in Experiments 1 and 2 for the 25 odorants.

#### Experimental procedure and EEG recordings

After reading instructions and providing written informed consent, subjects were seated in the test room and the experimenter fitted the EEG recording equipment onto the subject. Following skin cleaning (Skinpure; Nihon Kohden, Foothill Ranch, CA, USA) electrodes were attached to the scalp using Grass electrode paste (EC2; Grass, West Warwick, RI, USA). In order to cover frontal, central and parietal sites, in the left and right hemispheres and in the midline, EEG signals from Ag-AgCl electrodes were recorded at positions F3, Fz, F4, C3, Cz, C4, P3, Pz and P4 of the International 10/20 system (Brain Quick SD64 Micromed system amplifier), referenced to linked earlobes (A1+A2). Two additional electrodes were placed on the lateral canthus of the left eye and supra-ocularly to measure electro-ocular (EOG) activity. Eye blink artifact recordings larger than 50 µV were discarded. Impedance was kept below 5 kOhm. The sampling frequency was 256 Hz (bandpass 1–40 Hz). A ground electrode was placed on the forehead.

After fitting the physiological equipment, the experiment started. Each odorant trial was timed and cued by computer-generated voice instructions. The digitized voice prompted the subject to sniff as of a tone following a countdown (“three, two, one, sniff”). After each sniff, subjects rated odor intensity and pleasantness on a 1–9-point scale. There was no verbal interaction between experimenter and subject during odor presentation. Participants were instructed not to blink and to keep their eyes open during odor presentation.

The experiment was composed of 5 sessions, separated by 5 minutes pauses. Within session, each of the 20 odorants was presented once, interspersed with 10 blank trials (no odorant) in order to reduce adaptation. Thus, in total, each subject received 100 olfactory stimuli and EEG acquisition lasted approximately 120 minutes.

One important aspect of the experimental procedure was synchronization of odorant perception with EEG recording. Sampling instructions, odor presentation, sniffing and EEG recording were all time-locked via a single central computer. This was done by recording intra-nasal sniffing continuously during the experimental session. Sniffing was recorded using an airflow sensor (AWM720, Honeywell, France) connected to a nasal cannula positioned in each nostril. The sniffing signal was amplified and digitally recorded at 100 Hz using LabVIEW software®. This measurement was used to define the onset of each EEG trial and as a potential measure of interest in later analysis (i.e., comparing sniffing parameters across odorant conditions and age). Sniffs were pre-processed by removing baseline offsets and aligned in time by setting the point where the sniff entered the inspiratory phase as time 0. Inspired volume and sniff duration were calculated for each sniff and each participant. Both volume integration and sniff duration ended at the first data point at which the sniff returned to zero flow. Recording of respiratory data was linked to EEG recording by 1 TTL (transistor-transistor logic) pulse (5 V, negative to positive), ensuring accurate time-locking of all experimental trials.

#### Psychophysics data analysis

As in Experiment 1, for each participant, pleasantness estimates were averaged for all odorants of a given category, and served as a dependent variable in an ANOVA with “age” as a between-subjects factor (2: Young, Older) and “Hedonic valence” as a within-subject factor (2: pleasant, unpleasant). The same statistical analysis was done for intensity ratings. Significant level was set at p<.05 and all statistical analyses were performed using StatView® software.

#### EEG data analysis

All EEG analyses were performed using custom scripts written in Matlab (The MathWorks) and the Eeglab toolbox [53]. For OERSPs, the artifact-free signal was segmented from −1,000 to +1,200 ms around stimulus onset and each trial was analyzed in the time-frequency domain by convolution with complex Gaussian Morlet's wavelets. To remove ocular artefacts, an Independent Component Analysis (ICA) implemented in Eeglab was used. Moreover, each artefact-free epoch with amplitude values exceeding ±50 µV was rejected, ending in a rejection rate of 26% of the total number of epochs.

For each subject and for each electrode, signals were then averaged by “Conditions” (“no-odor”, “pleasant odors”, “unpleasant odors”) and “Age” (Young, Older). OERSPs were analyzed in a spectro-temporal window relative to baseline (time period: −1000 ms to 0 ms): from theta (θ: 4–8 Hz), alpha (α: 8–12 Hz), beta (β: 12–25 Hz), to low-gamma (γ-low: 25–35 Hz). The power (db) of the entire spectro-temporal window was averaged for each odorant condition and each subject, and was then compared between groups separately for the “no odor” condition, the “pleasant odors” condition, and the “unpleasant odors” condition using a statistical threshold of p<0.05 corrected for multiple comparisons (FDR).

Furthermore, activity within region of interest (ROI) was measured using the mean activity level inside a particular ROI.
